# Epigenetic regulation in cognitive impairment: Focus on N6‐methyladenosine modification and its potential role in perioperative neurocognitive disorders

**DOI:** 10.1002/ibra.70000

**Published:** 2025-09-11

**Authors:** Ting Liu, Xiao‐Juan Yang, Lin Zhou, Mi Gan, Ting‐Ting He, Sen Hong, Yan‐Yan Feng, Gao Su, You‐Xiao Zhao, Ying Cao, Qing‐Fan Zeng

**Affiliations:** ^1^ School of Anesthesiology Guizhou Medical University Guiyang China; ^2^ Department of Anesthesiology The Affiliated Jinyang Hospital of Guizhou Medical University/The Second People's Hospital of Guiyang Guiyang China; ^3^ Department of Anesthesiology The Affiliated Baiyun Hospital of Guizhou Medical University Guiyang China

**Keywords:** cognitive impairment, epitranscriptomics, N6‐methyladenosine, neuroinflammation, perioperative neurocognitive disorders

## Abstract

N6‐methyladenosine (m6A), the most abundant internal modification in mammalian mRNA, plays a critical role in cognitive function by dynamically regulating gene expression. This narrative review examines m6A's role in cognitive processes and its potential impact on perioperative neurocognitive disorders (PNDs), which encompass a spectrum including postoperative delirium, delayed neurocognitive recovery, and postoperative cognitive dysfunction. The m6A regulatory machinery—comprising methyltransferases (“writers”), demethylases (“erasers”), and recognition proteins (“readers”)—modulates neuronal development, synaptic plasticity, and cognitive processes by influencing mRNA stability, translation, and degradation. Evidence from animal models indicates that m6A dysregulation contributes to neuroinflammation, neurodegeneration, and neuronal injury, which are pathophysiological mechanisms implicated in PNDs. Notably, anesthetic agents and surgical stress have been shown to alter hippocampal m6A levels, and manipulation of m6A‐related proteins may ameliorate cognitive deficits. While these findings suggest potential mechanistic connections, direct evidence specifically linking m6A modifications to PNDs pathogenesis remains preliminary and largely based on preclinical models. Further research is needed to establish causal relationships, identify m6A‐modified targets relevant to PNDs pathology, and evaluate m6A as a potential biomarker or therapeutic target. This review provides a foundation for understanding how m6A modification may influence perioperative cognitive outcomes and identifies promising avenues for future investigation.

## Graphical abstract

N6‐methyladenosine (m6A), a key mammalian mRNA modification, affects cognition via gene regulation. This review explores its role in cognition and perioperative neurocognitive disorders (PNDs). Animal studies link m6A dysregulation to PND‐related mechanisms, but direct evidence is preliminary. More research is needed; the review guides future study.

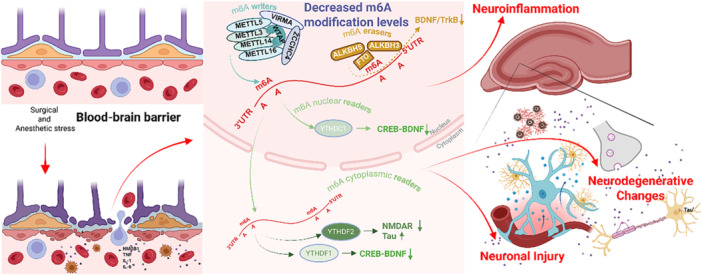

## 1 INTRODUCTION

Cognitive function disorders represent a significant challenge across various neurological conditions, with perioperative neurocognitive disorders (PNDs) emerging as a clinically important manifestation in surgical settings. PNDs encompass a spectrum of cognitive impairments during the perioperative period, constituting a prevalent complication of the central nervous system in surgical patients.^1^, ^2^ These disorders, classified according to their temporal presentation and clinical features, include preoperative cognitive decline, postoperative delirium (POD, typically onset within 1–3 days post‐surgery), delayed neurocognitive recovery (DNR, cognitive decline resolving by 30 days), and postoperative cognitive dysfunction (POCD, persistent impairment beyond 30 days). Beyond extending hospital stays and increasing healthcare expenditures, PNDs significantly compromise patients' quality of life and elevate the risk of long‐term cognitive deterioration and mortality, especially for older people.^3^, ^4^ While advanced age, pre‐existing neurodegenerative conditions, surgical trauma, anesthetic exposure, and inflammatory responses have been identified as primary risk factors,^5^, ^6^ the molecular mechanisms underlying these disorders remain incompletely characterized, highlighting the need for further investigation to develop effective preventive and therapeutic approaches.

Recent advances in epitranscriptomics have revealed the crucial role of RNA modifications in neural function, with N6‐methyladenosine (m6A) emerging as a key regulatory mechanism in gene expression. As the most abundant internal modification in mammalian mRNA, m6A dynamically influences mRNA processing, stability, translation, and degradation.^7^ This modification plays essential roles in neurodevelopment, synaptic plasticity, and memory formation, with its dysregulation linked to neurodegenerative conditions including Alzheimer's and Parkinson's diseases.^8^, ^9^ Importantly, the pathophysiological hallmarks of these disorders—neuroinflammation, neuronal injury, and synaptic dysfunction—also represent central mechanisms in PNDs pathogenesis.^10^ Evidence suggests that anesthetic agents and surgical stress can alter m6A methylation patterns in the hippocampus and other brain regions, potentially contributing to perioperative cognitive changes.^11^

Despite these connections, the relationship between m6A modification and PNDs remains underexplored. This narrative review addresses this knowledge gap by integrating current understanding of cognitive function, pathophysiology of PNDs, and m6A‐mediated gene regulation. We examine how m6A modifications influence cognitive processes broadly, with specific attention to their potential implications in perioperative settings. In this study, molecular mechanisms, and preclinical findings, we seek to elucidate how m6A modification might contribute to cognitive changes following anesthesia and surgery, and explore its potential as a biomarker or therapeutic target. It is important to note that the current evidence connecting m6A modification to PNDs exists at multiple levels of substantiation: (1) direct experimental evidence from animal models exposed to anesthetics and surgical stress, showing alterations in m6A patterns and cognitive outcomes; (2) indirect evidence from studies of m6A's role in related neurodegenerative and neuroinflammatory conditions that share pathophysiological features with PNDs; and (3) theoretical connections based on overlapping molecular pathways implicated in both m6A regulation and PNDs pathogenesis. While compelling, much of this evidence remains preliminary and is predominantly derived from preclinical models, necessitating cautious interpretation and further validation in clinical contexts. Enhanced understanding of this epitranscriptomic dimension may open new avenues for interventions to mitigate perioperative cognitive dysfunction, addressing an important clinical challenge in surgical and geriatric populations.

We performed a literature search using PubMed and Web of Science for English‐language articles up to March 2025, using keywords such as “postoperative delirium,” “postoperative cognitive dysfunction,” “perioperative neurocognitive disorders,” and “m6A RNA methylation.” Priority was given to high‐quality studies from the past 3 years (2020–2025) and relevant landmark studies. Additional references were identified from the bibliographies of pertinent articles to ensure comprehensive coverage of current evidence.

## 2 CHARACTERISTICS AND MECHANISMS OF PNDS

### 2.1 Characteristics and risk factors of PNDs

PNDs manifest as a continuum of cognitive impairments throughout the perioperative period, distinguished by their temporal profiles and clinical features.^12^ Among them, POD is characterized by acute, fluctuating disturbances in attention and consciousness post‐surgery, typically resolving within days, whereas POCD entails persistent deficits in memory, attention, and executive function, potentially becoming irreversible.^13^ POD represents the most common perioperative cognitive complication, with incidence significantly increasing among elderly patients and those undergoing complex surgical procedures. Notably, the epidemiology of PNDs varies significantly across surgical specialties, with particularly high incidence rates in certain populations. Cardiac surgery patients demonstrate the highest rates of PNDs, with POD affecting 4.1%–54.9% of patients.^14^ Elevated rates (20%–50%) are also observed following orthopedic surgeries, particularly hip fracture repairs.^15^, ^16^ While abdominal and neurosurgical procedures show generally lower POD incidence (approximately 5%–52% in abdominal surgeries and 12%–26% in neurosurgical procedures), PNDs still significantly impact recovery across these specialties.^17^, ^18^ Similarly, POCD occurs more frequently following cardiac surgeries and major operations, manifesting as long‐term cognitive impairment that substantially impacts quality of life^19^, ^20^ (Table 1).

**Table 1:** Characteristics and risk factors of PNDs.

Surgical specialty	POD incidence	POCD incidence	Key risk factors
Cardiac surgery	4.1%–54.9%^14^, ^21^	10%–60% at 3–6 months^22^ 10%–42% at 5 years^19^, ^20^, ^23^	CPB, diabetes, ICU stay duration cerebral hypoperfusion, microemboli, preoperative cognitive status, age > 65
Orthopedic surgery (hip fracture)	20%–50%^24^, ^25^, ^26^	15%–40% at 3 months^27^	Advanced age, preoperative cognitive impairment, pain management strategies, duration of surgery^15^, ^16^
Abdominal surgery	5%–52%^28^, ^29^	10%–30% at 3 months^30^, ^31^	Surgical duration, inflammatory response, age, preoperative comorbidities^17^, ^18^
Neurosurgery	4.2%–21.4%^32^, ^33^	20%–42% at 3 months^34^, ^35^	Direct brain manipulation, cerebral edema, intracranial pressure changes, age^17^
Vascular surgery	11.3%–35%^36^	10%–35% at 3 months^37^	Cerebral hypoperfusion, emboli, pre‐existing vascular disease
Minor procedures (e.g., ophthalmologic, dental)	4%–24%^38^	3%–6.6% at 3 months^39^, ^40^	Primarily influenced by patient‐related risk factors rather than procedure complexity

*Note*: Incidence rates vary significantly based on patient population characteristics (age, comorbidities, cognitive reserve), diagnostic criteria, assessment methods, and study timeframes. POCD incidence generally decreases over time, reflecting cognitive recovery in a subset of patients. Minor procedures include ophthalmologic, dental, and minor orthopedic or soft tissue surgeries.

Abbreviations: CPB, cardiopulmonary bypass; ICU, intensive care unit; POCD, postoperative cognitive dysfunction; POD, postoperative delirium.

**Figure 1:**
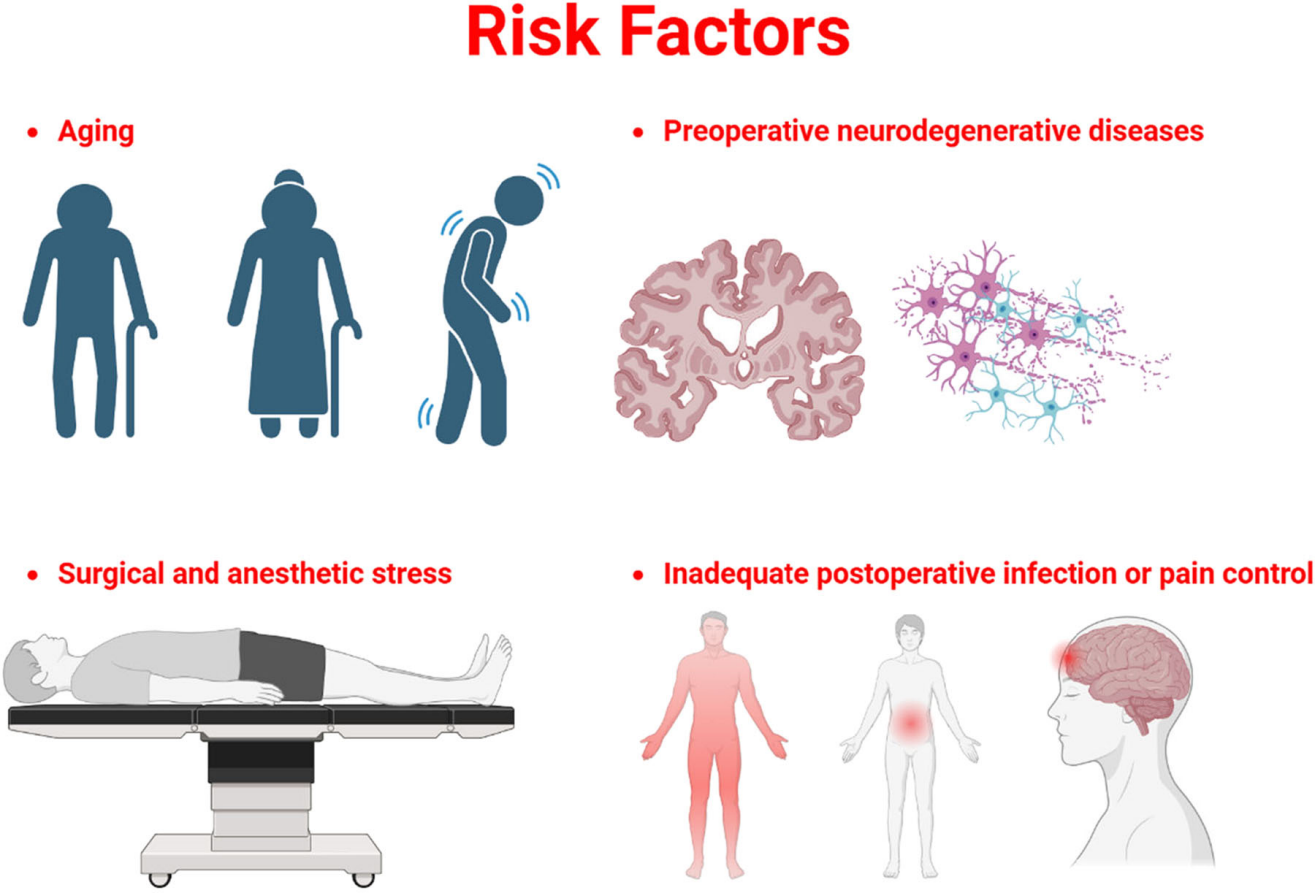
Predominant risk factors of perioperative neurocognitive disorders (PNDs). There are four major risk factors for PNDs, including aging, preoperative neurodegenerative diseases, surgical and anesthetic stress, and inadequate postoperative infection or pain control. [Color figure can be viewed at wileyonlinelibrary.com]

Predominant risk factors include preoperative conditions such as advanced age and prior cognitive impairment, intraoperative stressors like surgical duration and anesthesia type, and postoperative complications including infection and inadequate pain control.^41^, ^42^ These factors collectively amplify vulnerability, though exact interactions remain understudied, highlighting the multifactorial nature of PNDs and their impact on patient outcomes (Figure 1).

### 2.2 Temporal progression and diagnostic classification of PNDs

**Figure 2:**
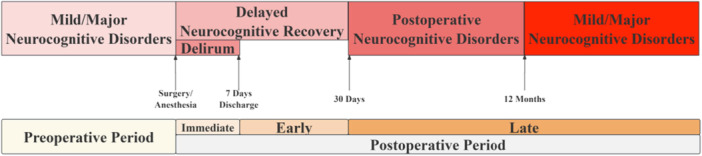
Temporal progression and diagnostic classification diagram of perioperative neurocognitive disorders (PNDs). PNDs follow a perioperative timeline and are divided into preoperative, immediate postoperative period (0–7 days), early postoperative period (up to 30 days), and late postoperative period (>30 days). [Color figure can be viewed at wileyonlinelibrary.com]

PNDs follow a distinct temporal progression that informs their classification and diagnosis based on the 2018 consensus nomenclature.^2^ This progression occurs along a perioperative timeline with specific diagnostic criteria for each phase (Figure 2). In the preoperative period, baseline cognitive assessment is essential to identify pre‐existing cognitive impairment, which represents both a risk factor and a diagnostic reference point for postoperative evaluation. Standardized neuropsychological testing typically includes assessments of memory, attention, executive function, and processing speed, establishing the foundation for detecting subsequent changes.

Following surgery, the immediate postoperative period (0–7 days) may feature POD, characterized by the acute onset of fluctuating attention, awareness, and cognition, typically developing within 1–3 days after surgery. Diagnosis relies on validated screening tools such as the Confusion Assessment Method, the Delirium Rating Scale‐Revised‐98, or the Intensive Care Delirium Screening Checklist. Key diagnostic features include disturbances in attention and awareness, cognitive changes, acute onset and fluctuating course, and evidence of an underlying medical condition, distinguishing this acute manifestation from other forms of PNDs.

As the patient continues recovery, the early postoperative period (up to 30 days) may reveal DNR, manifesting as cognitive decline that persists beyond the expected immediate postoperative period but resolves within 30 days. DNR is identified through neuropsychological testing, with a decline of at least 1–2 standard deviations from preoperative baseline in one or more cognitive domains. This transitional state bridges the gap between acute delirium and potential long‐term dysfunction, representing an important window for therapeutic intervention.

Beyond this recovery phase, the late postoperative period (beyond 30 days) may witness the development of POCD, presenting as persistent cognitive decline that extends beyond the 30‐day and potentially becomes permanent. POCD is diagnosed through comprehensive neuropsychological assessment batteries showing significant impairment compared to baseline and age‐matched controls. This chronic condition may affect multiple cognitive domains, with particular vulnerability in executive function, memory, attention, and information processing speed, indicating a more enduring disruption of neural networks that failed to recover despite the resolution of acute surgical stress.

This temporal classification has important implications for both research methodology and clinical management, contributing to cognitive assessments and interventions. Additionally, the progression from POD to POCD in some patients suggests potential shared underlying mechanisms that evolve over time, highlighting the importance of early identification and management of cognitive changes across the entire perioperative journey.

### 2.3 Pathophysiological mechanisms of PNDs

**Figure 3:**
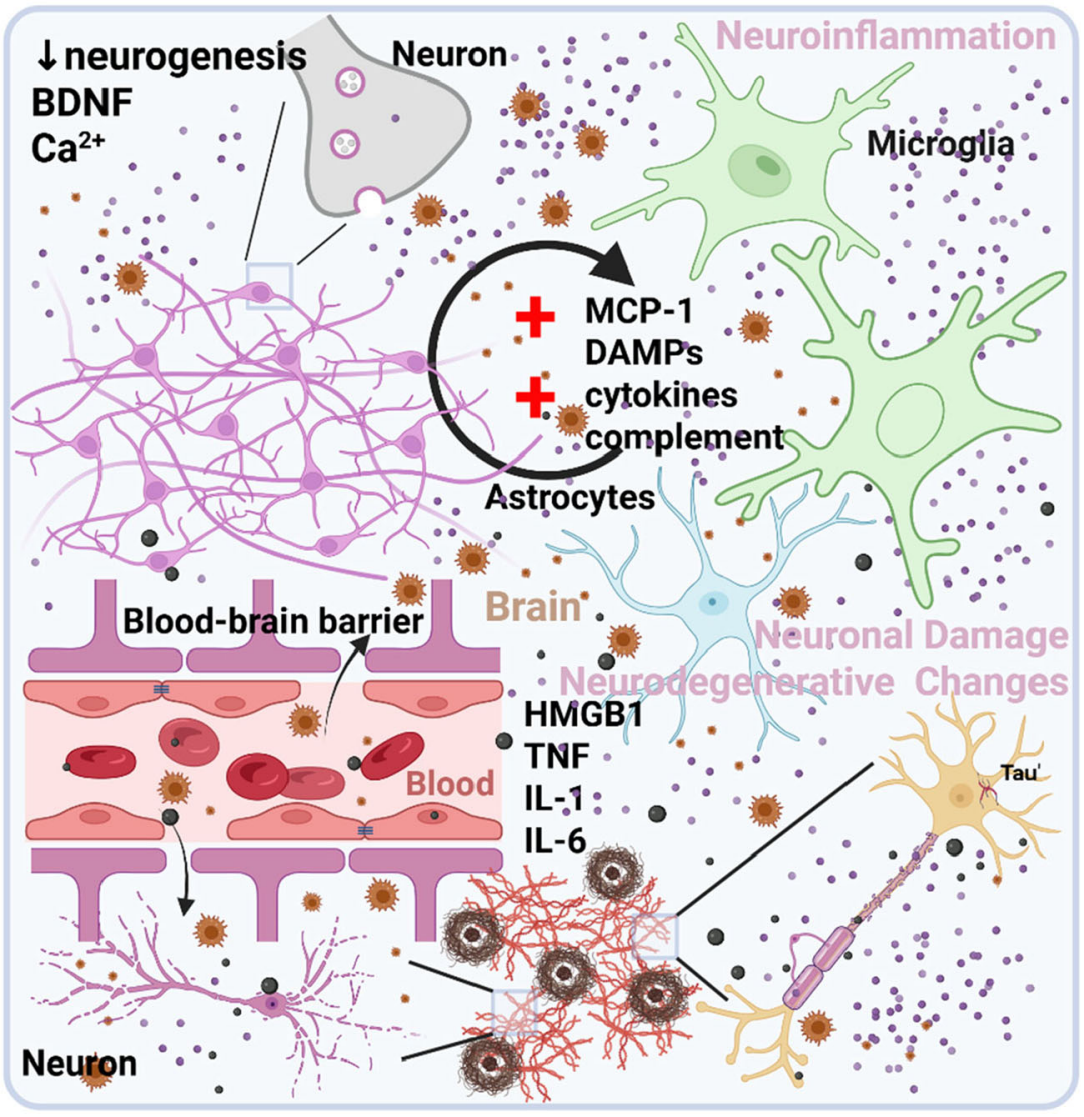
Pathophysiological mechanisms of perioperative neurocognitive disorders (PNDs). Three key pathophysiological mechanisms of PNDs include neuroinflammation, neurodegenerative changes, and neuronal damage. Various triggers initiate the release of inflammatory factors such as HMGB1, which cross the blood‐brain barrier into the central nervous system, activating microglia and producing pro‐inflammatory cytokines. This persistent inflammatory response leads to neuronal damage, synaptic dysfunction, and abnormal Tau protein aggregation, particularly pronounced in patients with pre‐existing neurodegenerative diseases. BDNF, brain‐derived neurotrophic factor; DAMPs, damage‐associated molecular patterns; HMGB1, high mobility group box chromosomal protein 1; IL‐1, interleukin‐1; IL‐6, interleukin‐6; MCP‐1, monocyte chemotactic protein 1; TNF, tumor necrosis factor. [Color figure can be viewed at wileyonlinelibrary.com]

The pathophysiology of PNDs centers on three interconnected core mechanisms: neuroinflammation, neurodegeneration, and neuronal damage, with inflammation serving as a key initiator.^43^ These mechanisms interact dynamically and synergistically to produce cognitive impairment, though their precise interactions remain incompletely resolved, necessitating targeted mechanistic studies (Figure 3).

#### 2.3.1 Neuroinflammation

Neuroinflammation drives PNDs pathogenesis, triggered by surgical trauma and anesthesia, with the release of high mobility group box 1 protein (HMGB1) as a pivotal initiating event.^44^ HMGB1, normally functioning as a nuclear protein, translocates extracellularly during perioperative stress, activating peripheral macrophages and monocytes to produce pro‐inflammatory cytokines such as interleukin‐1 (IL‐1), interleukin‐6 (IL‐6), and tumor necrosis factor.^45^ Experimental models demonstrate that sevoflurane anesthesia and surgery significantly elevate these inflammatory mediators alongside cognitive deficits,^46^ with increased HMGB1 expression detected in both peripheral tissues and hippocampal regions.^47^

POD can be considered a clinical manifestation of acute neuroinflammatory responses in the early postoperative period. Studies have documented significantly elevated levels of peripheral and central inflammatory markers in patients who develop POD.^48^, ^49^ This inflammatory state, when persistent, can trigger downstream pathological cascades leading to neuronal injury, synaptic dysfunction, and abnormal Tau protein aggregation. These changes provide a potential mechanism by which an acute delirium episode can progress to later cognitive dysfunction, as supported by data linking POD to subsequent POCD.^21^, ^46^

Blood‐brain barrier compromise allows peripheral inflammation to infiltrate the central nervous system, where sustained microglial activation amplifies inflammatory responses, impairing synaptic function and cognitive processes.^50^ This neuroinflammatory cascade establishes an important pathophysiological link between POD and POCD, with POD functioning as a significant risk factor for subsequent POCD development.^21^ Research has demonstrated that elevated early postoperative inflammatory markers, particularly IL‐6 and HMGB1, correlate significantly with long‐term cognitive deterioration,^46^ suggesting a continuous pathological process from acute to chronic cognitive impairment.

#### 2.3.2 Neurodegenerative changes

Advanced age, a primary risk factor, heightens the susceptibility to PNDs, likely due to diminished neuroplasticity and pre‐existing neurodegenerative changes.^18^ The 2018 consensus framework recognizes preoperative cognitive impairment—evidenced by lower baseline cognitive scores—as a significant predictor of POD, DNR, and POCD. Patients with conditions like Alzheimer's or Parkinson's disease exhibit elevated postoperative risks, reflecting underlying neurodegenerative vulnerabilities.^51^ The neuroinflammatory response triggered by surgery and anesthesia may accelerate these pre‐existing neurodegenerative processes, creating a pathological synergy that manifests as perioperative cognitive decline. Notably, although PNDs share pathophysiological features with Alzheimer's disease (such as neuroinflammation and Tau pathology), PNDs are distinguished by their acute onset following surgical stress and potential for recovery. Notably, m6A‐related mechanisms observed in Alzheimer's models—for instance, m6A‐facilitated clearance of pathological Tau^52^—may likewise modulate perioperative cognitive trajectories, but the transient nature of PNDs suggests some unique reversible elements compared to chronic neurodegeneration.

#### 2.3.3 Neuronal damage

Neuronal damage, often downstream of neuroinflammation, correlates with cognitive impairment, driven by surgery‐ or anesthesia‐induced apoptosis.^53^ Tau protein dysregulation, a marker of neurodegeneration, associates with PNDs severity, with sevoflurane exposure elevating Tau levels and impairing cognition in developmental models.^54^ A reduced beta‐amyloid/Tau ratio in cerebrospinal fluid (CSF) further signals increased PNDs risk.^55^ Brain‐derived neurotrophic factor (BDNF) reduction under surgical stress disrupts synaptic plasticity via tropomyosin receptor kinase B (TrkB) signaling, contributing to POCD.^56^ The interconnection between these mechanisms creates a complex pathophysiological cascade: perioperative stress triggers neuroinflammation, which, particularly in vulnerable patients with pre‐existing neurodegenerative changes, leads to neuronal damage and synaptic dysfunction. This integration of mechanisms helps explain the spectrum of cognitive impairments observed across different surgical populations and timeframes.

## 3 BIOLOGICAL PROPERTIES OF M6A MODIFICATION

### 3.1 Overview of m6A modification

M6A modification is the most prevalent internal modification in eukaryotic mRNA,^11^ regulates gene expression post‐transcriptionally by methylating adenosine at the N6 position, influencing protein synthesis pathways.^57^ Predominantly enriched near stop codons and 3' untranslated regions (3'UTRs) within RRACH consensus sequences (where R = G or A; H = A, C, or U),^58^, ^59^ m6A dynamically modulates multiple RNA metabolic processes. It alters RNA secondary structure to affect splicing through interactions with splicing factors,^60^ facilitates nuclear export via export receptors,^61^ enhances translation efficiency by promoting ribosome engagement,^62^ and regulates degradation by recruiting specific proteins.^63^ These regulatory processes are particularly pertinent to PNDs, where perioperative stress may disrupt neural gene expression, contributing to cognitive deficits. The brain exhibits among the highest m6A levels across mammalian tissues,^64^, ^65^ emphasizing its role in neural homeostasis and susceptibility to pathological disruption. This evolutionarily conserved modification varies across tissues, developmental stages, and physiological or pathological conditions,^66^, ^67^ underscoring its broad regulatory significance. Notably, these regulatory processes are also pertinent to PNDs, where perioperative stress may disrupt neural gene expression, contributing to cognitive deficits.

### 3.2 Regulatory mechanisms of m6A modification

m6A modification is governed by a dynamic interplay of methyltransferases (“writers”), demethylases (“erasers”), and recognition proteins (“readers”),^68^ adapting to diverse physiological and pathological contexts.^69^, ^70^, ^71^ This coordinated system enables precise control over RNA function and gene expression.

#### 3.2.1 m6A methyltransferases

Writers install m6A marks through a complex primarily comprising methyltransferase‐like 3 (METTL3), methyltransferase‐like 14 (METTL14), vir‐like m6A methyltransferase‐associated enzyme (VIRMA), and Wilms' tumor 1‐associated protein (WTAP).^72^, ^73^ METTL3, the catalytic core, utilizes S‐adenosylmethionine (SAM) to methylate RNA,^74^ enhancing m6A modification of inflammation‐related transcripts, such as HMGB1.^75^ METTL14 stabilizes METTL3, boosting its efficiency,^72^ with their knockdown reducing IL‐7 expression and inflammation.^76^ WTAP, guided by VIRMA,^77^ reinforces this complex,^78^ and its depletion markedly lowers m6A levels.^79^ WTAP also mitigates oxidative stress and apoptosis under inflammatory conditions via m6A regulation.^80^ Additional writers, including methyltransferase‐like 16 (METTL16),^81^ zinc finger CCHC‐type containing 4 (ZCCHC4),^82^ and methyltransferase‐like 5 (METTL5),^72^ diversify m6A deposition across RNA types.

#### 3.2.2 m6A demethylases

In contrast to writers, erasers remove m6A marks, enabling reversible regulation.^83^ Key erasers—fat mass and obesity‐associated protein (FTO), AlkB homolog 5 (ALKBH5), and AlkB homolog 3 (ALKBH3)^84^—belong to the Fe²⁺/α‐ketoglutarate‐dependent dioxygenase family.^85^ ALKBH5 and FTO display tissue‐specific activity,^86^, ^87^ with ALKBH5 reducing inflammation by upregulating Bcl‐2^88^ and its overexpression countering inflammatory responses.^89^, ^90^ FTO demethylates m6A via intermediates including N6‐hydroxymethyladenosine (hm6A) and N6‐ formyladenosine (f6A),^91^ and its knockout elevates m6A on TrkB mRNA, impairing neurotrophin signaling. High neural expression of FTO and ALKBH5 highlights m6A's role in brain function.^92^

#### 3.2.3 m6A recognition proteins

Readers interpret m6A marks to regulate downstream processes,^93^ including YTH domain family proteins (YTHDF1‐3, YTHDC1‐2), insulin‐like growth factor 2 mRNA‐binding proteins,^94^ and heterogeneous nuclear ribonucleoproteins (HNRNPC, HNRNPA2B1).^95^, ^96^ YTHDF1 enhances translation,^97^ YTHDF2 accelerates degradation and curbs inflammation via IL‐6R/JAK2/STAT1 inhibition,^98^ and YTHDF3 supports both.^99^, ^100^ YTHDC1 aids splicing and export, while YTHDC2 modulates stability and translation.^101^ This diversity enables fine‐tuned regulation,^89^, ^102^, ^103^ with readers implicated in neural development and pathology.^104^, ^105^, ^106^

## 4 ROLE OF M6A MODIFICATION IN THE PATHOPHYSIOLOGICAL MECHANISMS OF PNDS

### 4.1 m6A modification and neuroinflammation in PNDs

Neuroinflammation represents a fundamental driver in the pathogenesis of PNDs, with m6A modification playing a critical regulatory role in inflammatory responses that can either exacerbate or attenuate PNDs symptoms. The interplay between m6A‐related proteins and neuroinflammatory pathways demonstrates remarkable complexity across all components of the m6A regulatory machinery.

Writers such as METTL3 exhibit context‐dependent effects on neuroinflammation. In certain contexts, the METTL3/YTHDF1 complex enhances nuclear factor kappaB signaling by modifying m6A levels on nucleotide‐binding oligomerization domain 2 mRNA, triggering pro‐inflammatory cytokine release from microglia and amplifying neuroinflammation.^107^, ^108^ Conversely, METTL3 deficiency can heighten inflammation through dysregulation of HMGB1 expression,^109^ highlighting its dual regulatory potential in PNDs pathogenesis. The erasers of m6A marks similarly demonstrate complex roles in neuroinflammatory processes relevant to PNDs. ALKBH5 knockdown increases endoplasmic reticulum stress‐dependent neuroinflammation and neuronal apoptosis via the STAT5/PERK/EIF2α/CHOP pathway in a YTHDF1‐dependent manner.^110^ In traumatic brain injury models, FTO downregulation correlates with elevated pro‐inflammatory cytokine secretion,^111^ suggesting a potential protective role for FTO against excessive neuroinflammation in PNDs contexts.

The reader proteins that interpret m6A marks further modulate neuroinflammatory responses, with different readers exerting opposing effects. YTHDF1 promotes neuroinflammation by enhancing the translation of inflammatory mediators,^112^ while YTHDF2 mitigates inflammation by accelerating the degradation of inflammation‐related mRNAs and suppressing the IL‐6R/JAK2/STAT1 pathway.^113^

Animal studies further demonstrate that inflammatory dysregulation compromises blood‐brain barrier integrity, activates hippocampal microglia, and drives cognitive decline,^114^ collectively positioning m6A modification as a key modulator of neuroinflammation‐related PNDs.

### 4.2 m6A modification and neuronal injury in PNDs

Neuronal injury constitutes a defining feature of PNDs, with m6A modification intricately regulating neuronal survival, synaptic function, and axonal integrity.^115^ The relationship between m6A machinery and neuronal damage in PNDs contexts reveals several key mechanisms that may influence perioperative cognitive outcomes. In particular, Tau protein regulation represents a critical intersection between m6A modifications and neurodegeneration, causing neuronal injury. Tau dysregulation, common in neurodegenerative conditions and PNDs, is significantly influenced by m6A mechanisms. Studies in Alzheimer's disease models have shown that METTL3 stabilizes mRNAs to facilitate the clearance of phosphorylated Tau,^52^ and its overexpression in vivo reduces β‐amyloid‐induced synaptic damage and cognitive deficits.^116^ These findings suggest potential neuroprotective applications for modulating m6A writers in PNDs contexts, where similar pathological processes may contribute to cognitive impairment.

Beyond Tau regulation, neurotrophic signaling pathways are also subject to m6A‐mediated control with direct relevance to anesthetic‐induced neuronal injury. BDNF, vital for neuronal survival and synaptic plasticity, mediates sevoflurane‐induced neuronal injury and cognitive impairment through the cAMP/CREB pathway.^117^ Notably, FTO deficiency elevates m6A levels on the 3'UTR of TrkB mRNA, reducing its stability and expression, which disrupts the BDNF/TrkB signaling axis and leads to synaptic dysfunction and cognitive decline.^118^ This mechanism demonstrates how dysregulation of m6A erasers during perioperative stress might compromise neuronal resilience through altered neurotrophic support.

Together, these findings underscore m6A's pivotal role in linking epigenetic regulation to neuronal resilience and PNDs‐associated damage, offering potential therapeutic targets for intervention across multiple pathophysiological pathways.

### 4.3 m6A modification and neurodegenerative changes in PNDs

m6A modification‐related proteins connect surgical and anesthetic stress to neurodegenerative changes and cognitive dysfunction, primarily through their influence on key molecular pathways relevant to perioperative brain health. In sevoflurane‐induced POCD models, researchers have observed altered hippocampal m6A levels directly associated with cognitive impairment, providing direct evidence linking epitranscriptomic changes to anesthetic‐induced neurocognitive deficits. Importantly, YTHDF1 activation of the CREB/BDNF pathway ameliorates sevoflurane‐induced neuronal damage and cognitive dysfunction,^119^ directly implicating m6A “readers” not only in PNDs pathology but also as potential therapeutic targets.

The impact of surgical stress on m6A regulation further supports this connection, as experimental studies demonstrate that acute METTL3 knockout induces hippocampal cognitive deficits, highlighting its essential role in maintaining cognitive function during perioperative stress.^11^ This finding suggests that surgical procedures may disrupt normal m6A writer function, contributing to cognitive vulnerability in the perioperative period. Additionally, the activity of m6A demethylases appears particularly responsive to anesthetic exposure, as repeated sevoflurane exposure upregulates ALKBH5 in hippocampal tissue, and its inhibition with 5‐carboxy‐8‐hydroxyquinoline (IOX1) improves cognitive outcomes in these models,^120^ suggesting ALKBH5 as a potential therapeutic target in anesthetic‐induced cognitive dysfunction.

These interactive effects emphasize the multifaceted influence of m6A‐related proteins on neurodegenerative mechanisms in PNDs, revealing their dual potential as both pathogenic mediators and therapeutic targets depending on the specific context and regulatory network. Moreover, PNDs and Alzheimer's disease may converge on common m6A‐regulated pathways (such as Tau protein metabolis^115^, ^116^), even though PNDs unfolds acutely and may be partially reversible, unlike the progressive course of Alzheimer's disease. This mechanistic overlap provides valuable insights for therapeutic development that may eventually benefit patients with both perioperative and chronic neurodegenerative conditions.

**Figure 4:**
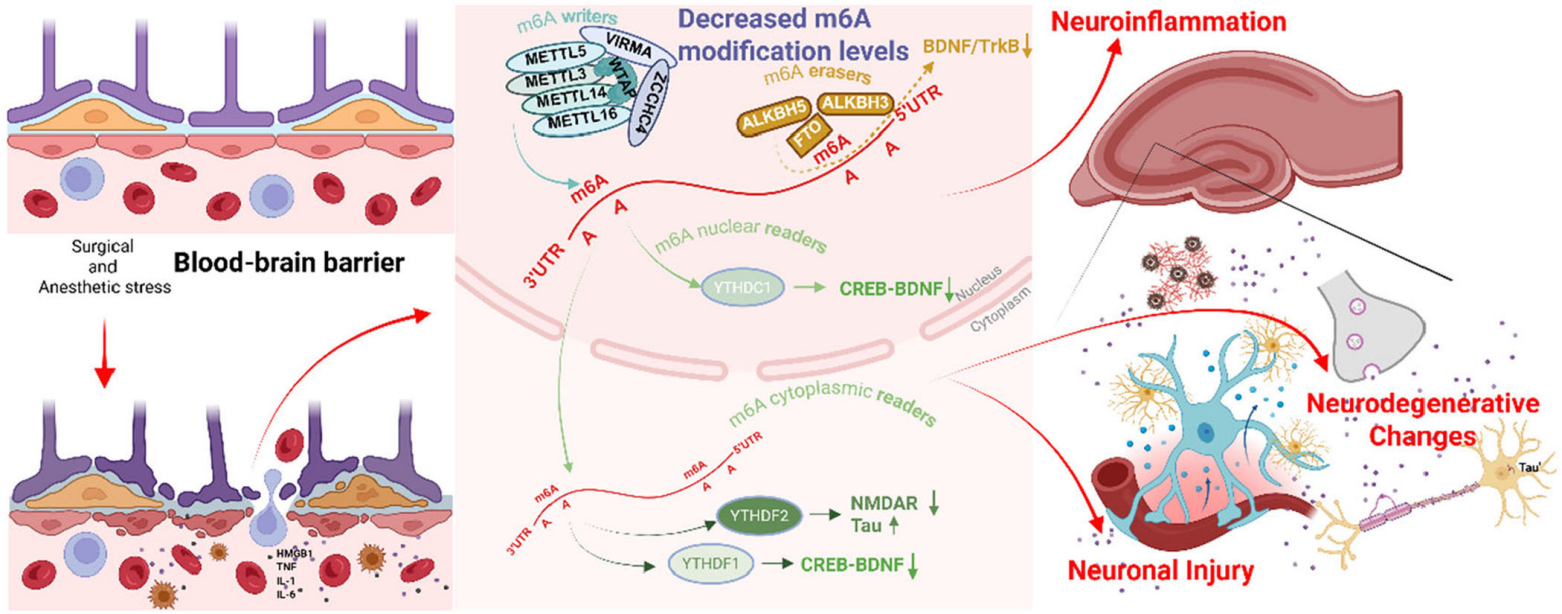
Role of m6A modification in the pathophysiological mechanisms of perioperative neurocognitive disorders (PNDs). Surgical and anesthetic stress compromise the blood‐brain barrier, allowing inflammatory factors (HMGB1, TNF, IL‐1, IL‐6) to enter the brain. The central portion depicts the m6A regulatory machinery, including writers (METTL3, METTL14, etc.), erasers (FTO, ALKBH5, etc.), and readers (YTHDF1, YTHDF2, YTHDC1, etc.). These modifications affect key pathways like CREB‐BDNF signaling and NMDAR/Tau regulation, ultimately contributing to the three major pathological outcomes of PNDs: neuroinflammation, neuronal injury, and neurodegenerative changes. ALKBH, AlkB homolog; BDNF, brain‐derived neurotrophic factor; CREB, cyclic AMP response element‐binding protein; FTO, fat mass and obesity‐associated protein; m6A, N6‐methyladenosine; METTL, methyltransferase‐like proteins; NMDAR, N‐methyl‐d‐aspartate receptor; TrkB, tropomyosin receptor kinase B; VIRMA, vir‐like m6A methyltransferase‐associated enzyme; WTAP, Wilms' tumor 1‐associated protein; YTHDF, YTH domain family proteins. [Color figure can be viewed at wileyonlinelibrary.com]

The potential role of m6A modification in the development of PNDs is significant, m6A‐related proteins exhibit complex interactions within neuroinflammatory pathways and contribute to neuronal injury. Furthermore, m6A modification affects neurodegenerative changes, notably through its regulation of Tau protein and its modulation of neurotrophic signaling, such as the BDNF/TrkB pathway. This modification might serve as a critical connection between epigenetic mechanisms and neuronal resilience, presenting itself as a valuable target for therapeutic strategies aimed at PNDs (Figure 4).

## 5 CURRENT LIMITATIONS AND FUTURE DIRECTIONS IN M6A‐PNDS RESEARCH

M6A modification emerges as a pivotal epigenetic regulator in PNDs, integrating neuroinflammation, neuronal injury, and neurodegenerative changes into a cohesive pathogenic framework. Despite significant progress, current research faces several notable challenges that must be addressed to advance clinical applications.

### 5.1 Limitations of current research

#### 5.1.1 Reliance on animal models

The majority of existing studies rely heavily on animal models, primarily rodents, where translating findings to human clinical contexts remains a critical barrier. As demonstrated, the use of sevoflurane‐induced cognitive impairment in rodent models, while informative, presents challenges in replicating the complex etiology of human PNDs.^121^ The inherent biological differences between species, particularly in terms of neural circuit organization and cognitive complexity, limit direct extrapolation to human pathophysiology. Furthermore, most animal models examine acute rather than long‐term effects of anesthetics on cognitive function, which may not adequately reflect the chronic nature of PNDs observed in clinical settings.

#### 5.1.2 Incomplete mechanistic understanding

The precise molecular mechanisms underlying m6A in PNDs remain incompletely understood. The current understanding of m6A modifications in PNDs lacks temporal and spatial specificity, with limited knowledge about how m6A patterns change dynamically during disease progression.^122^ Current research predominantly demonstrates correlative rather than causative relationships between m6A modifications and cognitive outcomes. Moreover, the cell‐type‐specific functions of m6A writers, erasers, and readers in different neural populations remain largely unexplored. The contradictory findings regarding whether specific m6A‐related proteins serve protective or pathogenic roles in different contexts further complicate mechanistic interpretations.

#### 5.1.3 Limited clinical evidence

Human studies investigating m6A modifications in PNDs contexts are scarce. As noted, while CSF and blood‐based biomarkers have been investigated in various neurocognitive disorders, the specific exploration of m6A modifications in patient samples from PNDs cases is remarkably limited.^123^ This creates a significant gap between preclinical findings and clinical applications. Additionally, the heterogeneity of PNDs manifestations in human populations—ranging from mild cognitive impairment to severe delirium—presents challenges in establishing consistent m6A‐related signatures across diverse patient cohorts.

#### 5.1.4 Methodological challenges

Technical limitations in accurately measuring dynamic m6A changes hamper progress in understanding context‐specific effects. Current m6A detection methods, including methylated RNA immunoprecipitation sequencing (MeRIP‐seq) and m6A‐RNA‐sequence technique (m6A‐seq), often lack the resolution to identify site‐specific modifications in low‐abundance transcripts that may be crucial for neural function. The lack of standardized protocols for measuring m6A levels in neural tissues further complicates cross‐study comparisons.^121^ Additionally, the complexity of isolating specific neural circuits relevant to PNDs from human brain samples presents substantial technical hurdles for translational research.

#### 5.1.5 Pharmacological specificity issues

As highlighted, current pharmacological tools targeting m6A machinery lack specificity, often affecting multiple RNA modifications simultaneously.^122^, ^124^ For instance, FTO inhibitors may affect both m6A and m6A modifications, complicating the interpretation of intervention studies. This lack of specificity hinders the precise manipulation of m6A pathways for both mechanistic studies and potential therapeutic applications in PNDs.

### 5.2 Future research directions

To address these limitations and advance the field, future research should prioritize several key directions:

#### 5.2.1 Clinical validation of m6A biomarkers

The establishment of reliable m6A‐related biomarkers represents a critical step toward clinical application in PNDs; longitudinal studies examining m6A markers (e.g., ALKBH5 expression patterns or global m6A profiles in blood and CSF) could yield valuable diagnostic and prognostic information when paired with standardized cognitive testing.^123^ Based on the identification of serum exosomal miRNAs as biomarkers, similar strategies could be used to quantify m6A‐modified transcripts in circulating exosomes as minimally invasive PNDs biomarkers. Machine learning algorithms may assist in identifying predictive m6A signatures to improve risk stratification for surgical patients. Additionally, establishing normal reference ranges for m6A levels across different ages and cognitive baselines would provide context for detecting pathological deviations in PNDs cases.

#### 5.2.2 Mechanistic clarification

Employing multi‐omics approaches, including integrating transcriptomics, proteomics, and epitranscriptomics data, represents a promising strategy to unravel how m6A modifications interact with other regulatory mechanisms under perioperative stress.^124^ Single‐cell sequencing of vulnerable brain regions (e.g., hippocampus and prefrontal cortex) should be leveraged to delineate cell‐type‐specific m6A patterns during PNDs development. It is also important to determine how anesthetic agents and surgical stress modulate m6A machinery and interact with inflammatory mediators. Advanced techniques like CRISPR‐Cas13‐based RNA editing enable precise manipulation of m6A marks to establish causative links between specific modifications and cognitive outcomes. Furthermore, developing temporally controlled, cell‐type‐specific conditional knockout models for m6A‐related proteins will clarify their roles in different phases of PNDs progression. Finally, investigations should examine the influence of aging, pre‐existing cognitive impairment, and other comorbidities on m6A‐mediated vulnerability, addressing the clinical complexity of these risk factors.

#### 5.2.3 Therapeutic development

Preclinical results suggest that targeting m6A pathways could be a viable therapeutic strategy. For instance, Meng et al. showed that the ALKBH5 inhibitor IOX1 improved cognition in a sevoflurane‐induced POCD model.^124^ Future drug development should prioritize selectivity—designing compounds that specifically target individual m6A writers, erasers, or readers with minimal off‐target effects. High‐throughput screening and structure‐based drug design approaches can accelerate the discovery of m6A modulators optimized for central nervous system delivery. In particular, developing antagonists that block reader proteins from recognizing pathogenic m6A‐marked transcripts may offer more precise intervention than broadly inhibiting writers or erasers. Combination therapies also merit exploration. For example, pairing an m6A modulator with an anti‐inflammatory or neuroprotective agent could address multiple aspects of PNDs pathophysiology. Additionally, nonpharmacological interventions such as transcranial magnetic stimulation or cognitive training might indirectly modulate m6A pathways and serve as complementary strategies to enhance brain resilience.

#### 5.2.4 Translational models and approaches

Developing more human‐relevant translational models that better recapitulate human PNDs pathophysiology is essential. For instance, human‐induced pluripotent stem cell‐derived neural organoids exposed to clinically relevant anesthetic regimens could bridge the gap between rodent studies and human biology, enabling the testing of m6A‐targeted interventions in a human neural tissue context before clinical trials. In parallel, establishing international collaborative networks to collect and analyze patient samples across diverse surgical populations would provide the statistical power needed to detect m6A‐related biomarkers amid the heterogeneity of PNDs presentations. Standardized protocols for tissue collection, processing, and m6A analysis across centers will be critical to ensure multi‐center data comparability.

#### 5.2.5 Preventive strategies based on m6A insights

Insights from m6A research could inform strategies to prevent PNDs in high‐risk patients. For example, preoperative cognitive enrichment (training) or pharmacological preconditioning targeting m6A pathways might enhance neural resilience before surgical stress.^125^, ^126^ Additionally, developing personalized anesthetic approaches based on an individual's m6A epitranscriptomic profile could help minimize cognitive risk in vulnerable populations (such as older patients or those with pre‐existing cognitive impairment).

This integrated approach aims to bridge the translational divide between preclinical discoveries and clinical applications, ultimately leading to novel diagnostic tools, preventive strategies, and therapeutic interventions for PNDs based on m6A modification pathways.

## AUTHOR CONTRIBUTIONS

Ying Cao and Qing‐Fan Zeng conceptualized and got administrative support. Ting Liu and Xiao‐Juan Yang wrote, revised, and edited the manuscript. Lin Zhou, Mi Gan, Ting‐Ting He, Sen Hong, Yan‐Yan Feng, Gao Su, and You‐Xiao Zhao collected material and composed the paper. Lin Zhou assisted with language polish and figure modifications. All authors read and approved the final content of this manuscript.

## CONFLICT OF INTEREST STATEMENT

The authors declare no conflicts of interest.

## ETHICS STATEMENT

This review article is based on previously published literature and does not involve primary research with human subjects, animal experiments, or other activities requiring ethical approval. All cited sources have been appropriately referenced and acknowledged. The authors declare no ethical conflicts in the preparation of this manuscript.

## Data Availability

The manuscript does not contain novel data, and all the information provided can be sourced from literature.
